# Is Radical Surgery Alone Enough in T1-3N1a Colon Cancer?

**DOI:** 10.3389/fonc.2020.01679

**Published:** 2020-10-22

**Authors:** Guoxiong Xu, Yiqi Jin, Changwen Fang, Jingfan Yu, Zhixuan Zhang, Chunrong Sun

**Affiliations:** Department of General Surgery, The Affiliated Suzhou Hospital of Nanjing Medical University, Suzhou Municipal Hospital, Suzhou, China

**Keywords:** radical surgery, adjuvant chemotherapy, colon cancer, lymph node, burden

## Abstract

**Background:** Low lymphatic tumor burden is associated with a better prognosis. However, it is uncertain whether those patients diagnosed as cN0 found to be pN+ could be a favorable subgroup in stage III disease. Radical surgery alone might avoid overtreatment in those patients.

**Methods:** Eligible patients diagnosed with colon cancer without metastasis were recruited from the Surveillance, Epidemiology, and End Results (SEER) database from 2004 to 2016 using SEER^*^Stat 8.3.5 software (Surveillance Research Program, National Cancer Institute) and divided into two groups: surgery group (*n* = 3,081) and surgery followed by adjuvant chemotherapy group (*n* = 4,591). Overall survival (OS) and cause-specific survival (CSS) differences were assessed by Kaplan–Meier analysis, and survival differences were estimated with log-rank tests. Univariate and multivariate Cox proportional hazard regressions were used to assess hazard ratios (HRs) and 95% confidence intervals (CIs) for colon cancer patients.

**Results:** A total of 7,672 pT1-3N1a colon cancer patients were recruited from 208,751 colon cancer patients. The 5-year CSS rates of patients without and with adjuvant chemotherapy were 80.0 and 90.7%, respectively. The receipt of adjuvant chemotherapy after the radical resection of the primary tumor was independently associated with 57.3% decreased risk of colon cancer-specific mortality compared with surgery alone (HR = 0.427, 95% CI = 0.370–0.492, *P* < 0.001, using surgery alone as the reference).

**Conclusions:** Adjuvant chemotherapy was significantly associated with improved prognosis and radical surgery alone did not provide enough treatment for colon cancer with very low lymphatic tumor burden.

## Background

Colorectal cancer (CRC) is one of the most common cancers and among the leading causes of cancer-related mortality worldwide (1–3). Lymph node status is the most important prognostic factor in non-metastatic colon cancer (4–6). It has been estimated that the sensitivity of nodal involvement in colon cancer with preoperative CT was 71% (95% CI, 59–81%), which indicates that ~30% of lymph node involvement is missed due to the normal size (caused by very low lymphatic tumor burden) and because it is only revealed in the pathological report after surgery (7). Previous research has suggested that T4N0 colon cancer patients have inferior 5-year overall survival (5-OS) compared with T1-2N1 patients (8–10). A low lymphatic tumor burden could be associated with a better prognosis compared to the higher T stage without lymph node metastasis. However, it remains uncertain whether those with clinically node-negative colon cancer and pathologically diagnosed node involvement could be a favorable subgroup in stage III disease. This study explores this, examining whether this subgroup of colon cancer patients could be treated with radical surgery alone to avoid overtreatment.

## Materials and Methods

### Database and Study Population

**Figure 1:**
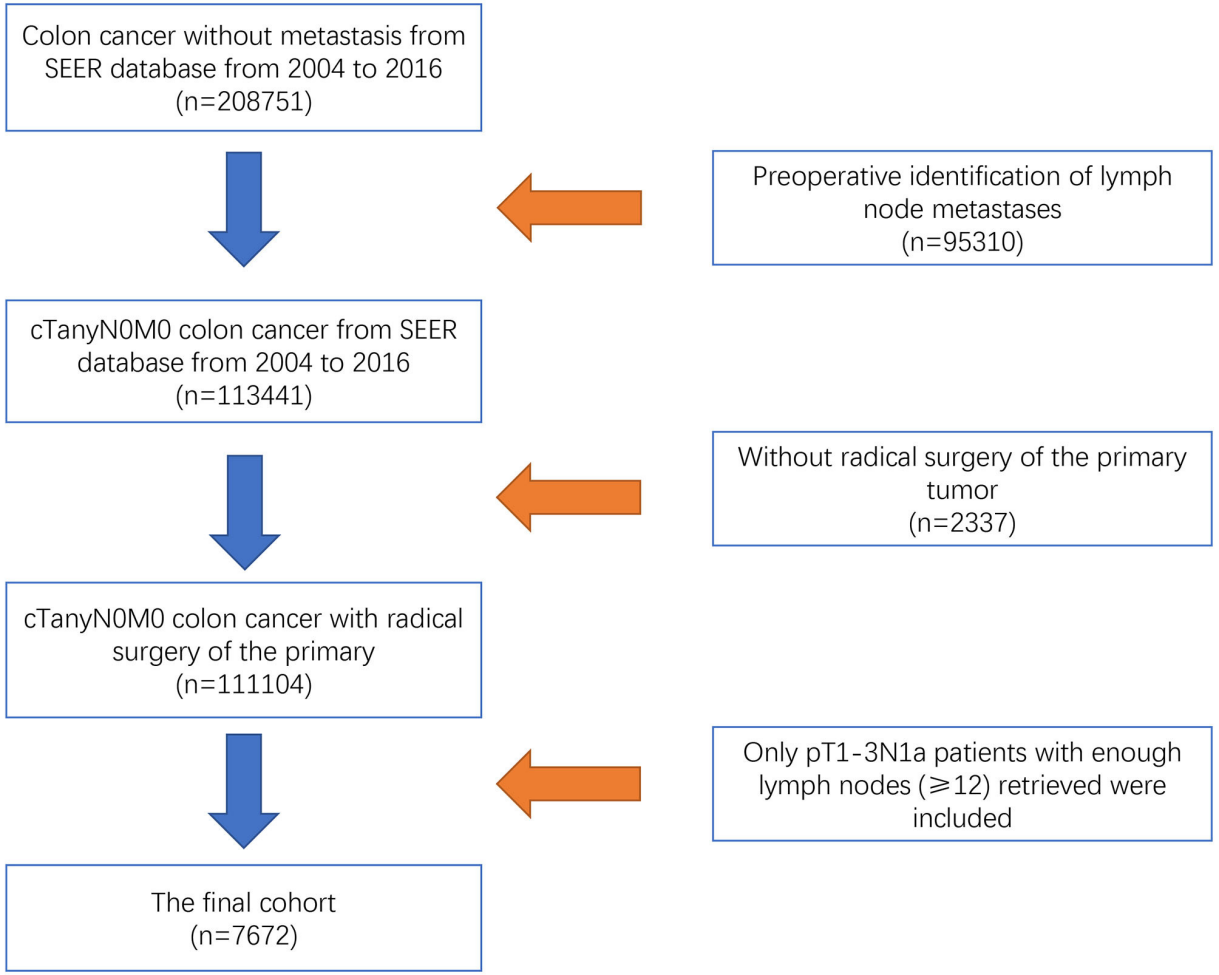
Flow chart of the selection process for pT1-3N1a colon cancer patients from the SEER database.

As a public database with free access, the Surveillance, Epidemiology, and End Results (SEER) program of the National Cancer Institute (NCI) covers ~27.8% of cancer cases in the United States. Using SEER^*^Stat 8.3.5 software (Surveillance Research Program, National Cancer Institute), we collected data from patients who were diagnosed with colon cancer without metastasis from the SEER database from 2004 to 2016. Patients without complete information on the TNM stage or active follow-up were excluded from the study. We also excluded patients with preoperative identification of lymph node metastases (*n* = 95,310) or who did not receive radical surgery of the primary tumor (*n* = 2,337). The efficacy of adjuvant chemotherapy in T4 disease has been confirmed in recent studies (11–13). For example, Kumar et al. (12) found that the survival benefits of adjuvant chemotherapy were mainly observed in patients with T4 disease compared with other high-risk factors. This study aimed to investigate whether radical surgery alone was enough in colon cancer with a very low lymphatic tumor burden, the study subjects were focused on colon cancer with only one lymph node metastasis by postoperative pathologic results (pN1a), which was not confirmed by preoperative examination. Therefore, only pT1-3N1a colon cancer patients from whom enough lymph nodes (≥12) were retrieved are included in our analyses (*n* = 7,672, Figure 1). For the final cohort, we divided patients into two groups: surgery group (*n* = 3,081) and surgery followed by adjuvant chemotherapy group (*n* = 4,591). The continuous variables were transformed into categorical variables based on recognized cut-off values. The relevant variable definitions and information including T stage (including T1, T2, and T3), age (years), race/ethnicity (including white, black, and other), gender (including male and female), grade (including grade I/II, grade III/IV, and unknown), and histological type (including adenocarcinoma, and mucinous adenocarcinoma/signet ring cell carcinoma) were extracted from the SEER database.

### Statistical Analysis

In our analyses, the Chi-square test was performed to compare categorical variables between patients in the surgery group and surgery and adjuvant chemotherapy group. The outcomes of interest included overall survival (OS) and cause-specific survival (CSS). OS and CSS differences were assessed by Kaplan–Meier analysis, and survival differences were estimated with log-rank tests. Univariate and multivariate Cox proportional hazard regressions were used to assess hazard ratios (HRs) and 95% confidence intervals (CIs) of patient characteristics for pT1-3N1a colon cancer patients. Only clinicopathologic characteristics that showed prognostic significance (log rank, *P* < 0.20) in univariate Cox analyses were entered in multivariate Cox analyses. For CSS, these prognostic factors included the receipt of chemotherapy, T stage, age, race/ethnicity, grade, and histological type; for OS, these prognostic factors included the receipt of chemotherapy, T stage, age, race/ethnicity, gender, grade, and histological type. All tests were two sided, and two sided *P* < 0.05 was considered statistically significant in our analyses. All analyses were conducted using SPSS version 23 statistical software (IBM Corporation).

## Results

### Patient Clinicopathological Characteristics

A total of 7,672 pT1-3N1a colon cancer patients who met with the strict inclusion criteria of our analyses, were recruited from 208,751 colon cancer patients included on the SEER database between 2004 and 2016, including 3,714 male (48.4%) and 3,958 female (51.5%) patients. Among them, 238 (7.7%) patients were T1 stage, 467 (15.2%) patients were T2 stage, and 2,376 (77.1%) patients were T3 stage. The mean age was 69 years. Clinicopathologic characteristics of the whole cohort regarding the receipt of chemotherapy, T stage, age, race/ethnicity, gender, grade, and histological type are listed in Table 1. The median follow-up durations were 42 months. The 3 and 5-year CSS rates in the SEER cohort were 91.0 and 86.6%, respectively. The 3 and 5-year OS rates in the SEER cohort were 79.2 and 69.9%, respectively.

**Table 1:** Clinical characteristics of two groups of patients included in the final study cohort.

**Variables**	**Number (%)**		***P***
	**Surgery** **(*N* = 3081)**	**Surgery and adjuvant chemotherapy** **(*N* = 4591)**	
T stage			0.001
T1	238 (7.7)	463 (10.1)	
T2	467 (15.2)	739 (16.1)	
T3	2,376 (77.1)	3,389 (73.8)	
Age (years)			<0.001
≤ 65	670 (21.7)	2,522 (54.9)	
>65	2,411 (78.3)	2,069 (45.1)	
Race			0.070
White	2,444 (79.3)	3,545 (77.2)	
Black	391 (12.7)	623 (13.6)	
Other	246 (8.0)	423 (9.2)	
Gender			0.024
Male	1,443 (46.8)	2,271 (49.5)	
Female	1,638 (53.2)	2,320 (50.5)	
Grade			0.007
Grade I/II	2,407 (78.1)	3,718 (81.0)	
Grade III/IV	626 (20.3)	801 (17.4)	
Unknown	48 (1.6)	72 (1.6)	
Histological type			0.149
Adenocarcinoma	2,806 (91.1)	4,224 (92.0)	
Mucinous adenocarcinoma/signet ring cell carcinoma	275 (8.9)	367 (8.0)	

As shown in Table 1, the T3 stage was less likely to receive adjuvant chemotherapy (*P* = 0.001); older patients were less likely to receive adjuvant chemotherapy (*P* < 0.001); male patients were more likely to receive adjuvant chemotherapy (*P* = 0.024).

### The Survival Benefits of Adjuvant Chemotherapy in pT1-3N1a Colon Cancer Patients

**Figure 2:**
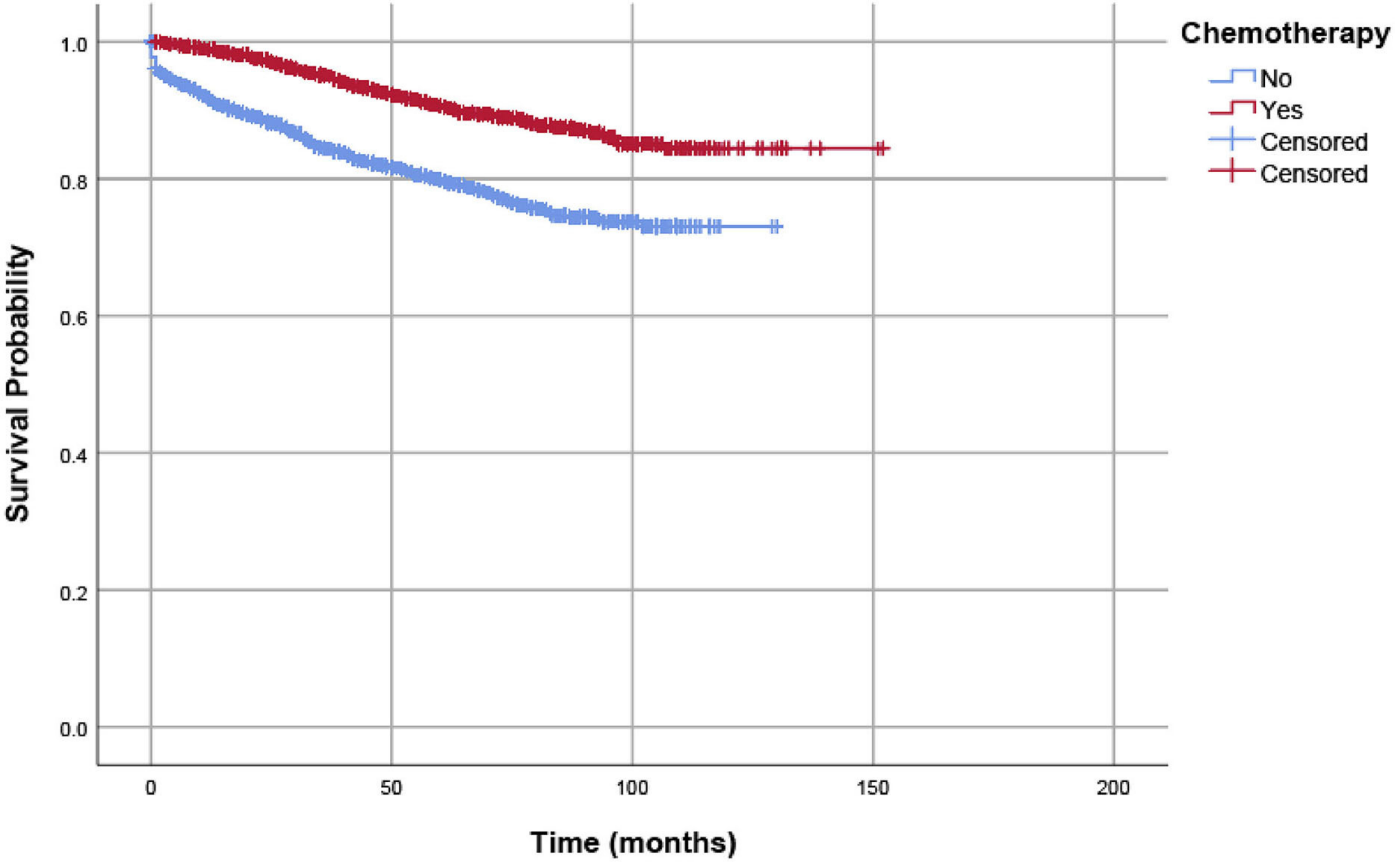
CSSs of eligible patients were assessed according to the receipt of chemotherapy by Kaplan–Meier analysis, and survival differences were estimated with log-rank tests.

In our analyses, OS and CSS were assessed by Kaplan–Meier analysis, and survival differences were estimated with log-rank tests. As shown in Figure 2, in pT1-3N1a colon cancer patients without preoperative identification of lymph node metastases, adjuvant chemotherapy was significantly associated with improved CSS. The 5-year CSS rates of patients without adjuvant chemotherapy and patients with adjuvant chemotherapy were 80.0 and 90.7%, respectively.

We used Cox proportional hazard regression analyses to evaluate potential risk factors and the efficacy of adjuvant chemotherapy. Table 2 shows the results of univariate and multivariate Cox regression analyses for CSS in the whole cohort. Clinicopathologic characteristics that showed prognostic significance (log rank, *P* < 0.20) in univariate Cox analyses were entered in multivariate Cox analyses, including the receipt of adjuvant chemotherapy, T stage, age (years), race/ethnicity, grade, and histological type. In multivariate Cox analyses, higher T stage was associated with increased risk of colon cancer-specific mortality (HR = 1.570, 95% CI = 1.019–2.419, *P* =0.041 for T2 stage; HR = 3.320, 95% CI = 2.272–4.852, *P* < 0.001 for T3 stage; using T1 stage as the reference). Older patients were also associated with increased risk of colon cancer-specific mortality (HR = 1.381, 95% CI = 1.186–1.608, *P* < 0.001, using ≤ 65 years as the reference), and people of black ethnicity are also associated with an increased risk of colon cancer-specific mortality (HR = 1.451, 95% CI = 1.210–1.740, *P* < 0.001, using white ethnicity as the reference). More importantly, the receipt of adjuvant chemotherapy after the radical resection of the primary tumor was independently associated with 57.3% decreased risk of colon cancer-specific mortality compared with surgery alone (HR = 0.427, 95% CI = 0.370–0.492, *P* < 0.001, using surgery alone as the reference).

**Table 2:** Univariate and multivariate Cox regression analyses for CSS in the whole cohort.

**Variable**	**Univariate analysis**		**Multivariate analysis**	
	**HR (95%CI)**	***P***	**HR (95%CI)**	***P***
Group		<0.001		<0.001
Surgery	1		1	
Surgery and adjuvant chemotherapy	0.386 (0.337–0.442)		0.427 (0.370–0.492)	
T stage		<0.001		<0.001
T1	1		1	
T2	1.703 (1.107–2.621)		1.570 (1.019–2.419)	0.041
T3	3.620 (2.483–5.279)		3.320 (2.272–4.852)	<0.001
Age (years)		<0.001		<0.001
≤ 65	1		1	
>65	1.845 (1.598–2.132)		1.381 (1.186–1.608)	
Race		<0.001		<0.001
White	1		1	
Black	1.343 (1.121–1.608)		1.451 (1.210–1.740)	<0.001
Other	0.767 (0.586–1.006)		0.817 (0.623–1.071)	0.144
Gender		0.729		
Male	1			
Female	0.977 (0.855–1.116)			
Grade		0.039		0.416
Grade I/II	1		1	
Grade III/IV	1.233 (1.048–1.451)		1.111 (0.943–1.309)	0.209
Unknown	0.942 (0.532–1.667)		1.159 (0.652–2.059)	0.615
Histological type		0.128		0.622
Adenocarcinoma	1		1	
Mucinous adenocarcinoma/signet ring cell carcinoma	1.194 (0.950–1.501)		1.060 (0.841–1.335)	

**Figure 3:**
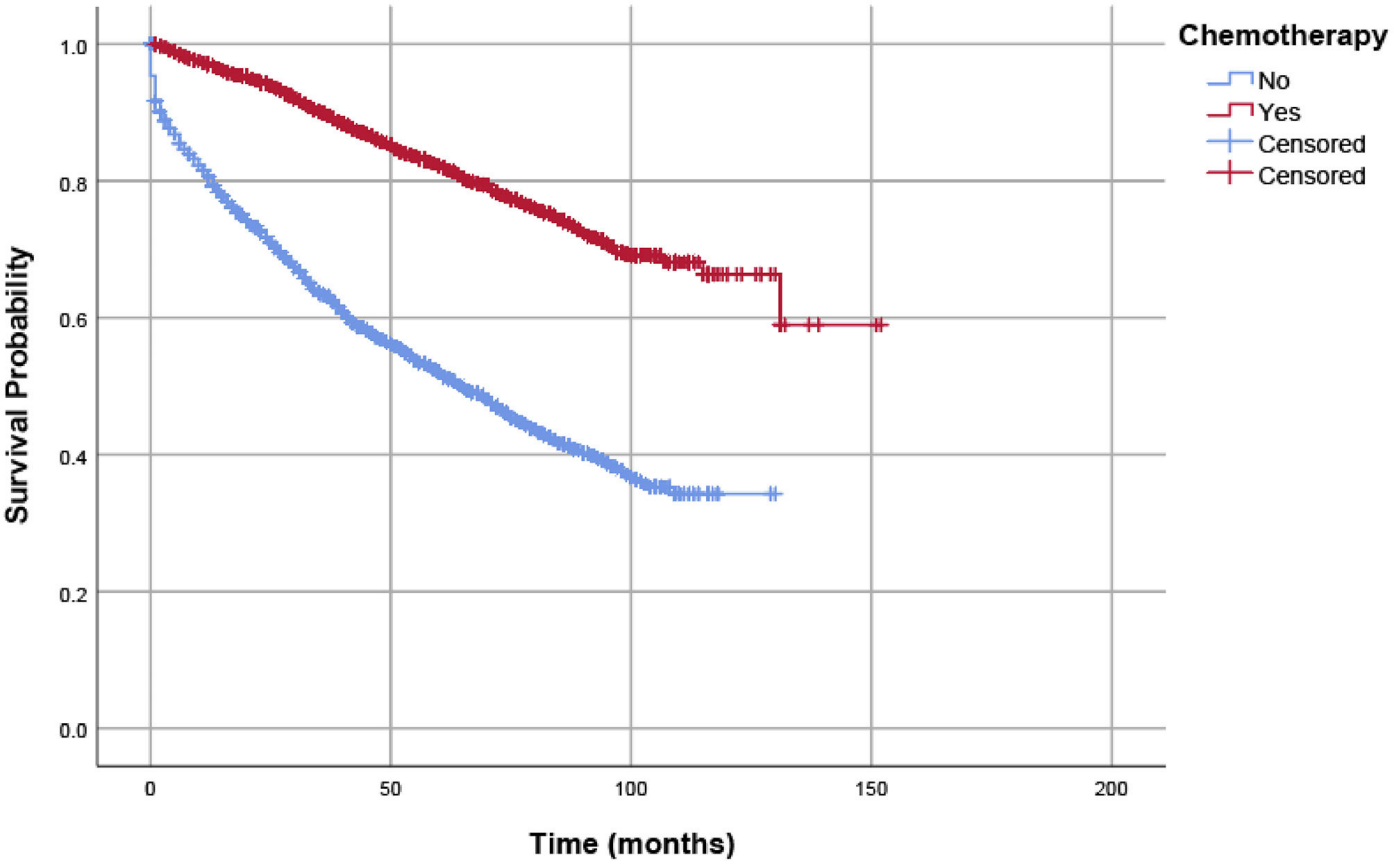
OSs of eligible patients were assessed according to the receipt of chemotherapy by Kaplan–Meier analysis, and survival differences were estimated with log-rank tests.

The study also used OS as the endpoint to evaluate the prognostic value of clinicopathologic features in the whole cohort. This also found that adjuvant chemotherapy was significantly associated with improved OS. The 5-year OS rates of patients without and with adjuvant chemotherapy were 52.1 and 82.1%, respectively (Figure 3). Table 3 shows the results of univariate and multivariate Cox regression analyses for OS in the whole cohort. Clinicopathologic characteristics that showed prognostic significance (log rank, *P* < 0.20) in univariate Cox analyses were entered in multivariate Cox analyses, including the receipt of adjuvant chemotherapy, T stage, age (years), race/ethnicity, gender, grade, and histological type. In multivariate Cox analyses, higher T stage was associated with increased risk of overall mortality (HR = 1.219, 95% CI = 0.985–1.508, *P* = 0.068 for T2 stage; HR = 1.688, 95% CI = 1.403–2.032, *P* < 0.001 for T3 stage; using T1 stage as the reference), older patients were associated with increased risk of overall mortality (HR = 2.262, 95% CI = 2.034–2.517, *P* < 0.001, using ≤ 65 years as the reference), and black ethnicity was associated with increased risk of overall mortality (HR = 1.187, 95% CI = 1.049–1.344, *P* = 0.007, using white ethnicity as the reference), it was also found that gender was significant, and female patients were associated with a decreased risk of overall mortality (HR = 0.848, 95% CI = 0.779–0.922, *P* < 0.001, using male gender as the reference), and mucinous adenocarcinoma/signet ring cell carcinoma was associated with increased risk of overall mortality (HR = 1.212, 95% CI = 1.056–1.391, *P* = 0.006, using adenocarcinoma as the reference). The receipt of adjuvant chemotherapy after the radical resection of the primary tumor was also shown to be independently associated with a 64.0% decreased risk of overall mortality compared with surgery alone (HR = 0.360, 95% CI = 0.329–0.394, *P* < 0.001, using surgery alone as the reference).

**Table 3:** Univariate and multivariate Cox regression analyses for OS in the whole cohort.

**Variable**	**Univariate analysis**		**Multivariate analysis**	
	**HR (95%CI)**	***P***	**HR (95%CI)**	***P***
Group		<0.001		<0.001
Surgery	1		1	
Surgery and adjuvant chemotherapy	0.289 (0.265–0.316)		0.360 (0.329–0.394)	
T stage		<0.001		<0.001
T1	1		1	
T2	1.417 (1.147–1.752)	0.001	1.219 (0.985–1.508)	0.068
T3	1.990 (1.656–2.390)	<0.001	1.688 (1.403–2.032)	<0.001
Age (years)		<0.001		<0.001
≤ 65	1		1	
>65	3.126 (2.823–3.462)		2.262 (2.034–2.517)	
Race		<0.001		<0.001
White	1		1	
Black	1.029 (0.910–1.164)	0.649	1.187 (1.049–1.344)	0.007
Other	0.687 (0.577–0.817)	<0.001	0.749 (0.630–0.892)	0.001
Gender		0.051		<0.001
Male	1		1	
Female	0.920 (0.847–1.000)		0.848 (0.779–0.922)	
Grade		0.001		0.245
Grade I/II	1		1	
Grade III/IV	1.207 (1.090–1.338)	<0.001	1.058 (0.954–1.173)	0.286
Unknown	1.193 (0.869–1.639)	0.275	1.250 (0.907–1.721)	0.172
Histological type		<0.001		0.006
Adenocarcinoma	1		1	
Mucinous adenocarcinoma/signet ring cell carcinoma	1.352 (1.180–1.549)		1.212 (1.056–1.391)	

## Discussion

Similar to cases with rectal cancer, the preoperative diagnosis of lymph node positivity with CT (computerized tomography) in colon cancer was a problem for radiologists, and false-negative results were usually caused by microscopic metastatic lymph nodes with a normal size (7). However, node-negative colon cancers that were not clinically visible on preoperative imaging also indicated very low lymphatic tumor burdens. As we already know, the overuse of chemotherapy increases economic burdens because of the high cost of chemotherapy and has adverse effects on the personal lives and work of patients (14, 15). With this in mind, this study aimed to explore whether those with clinically node-negative colon cancer, pathologically diagnosed with node involvement, could be a favorable subgroup in a study of stage III disease and whether they could be treated with radical surgery alone to avoid overtreatment. The investigation of this question is of clinical significance, and to the best of our knowledge, has not been specifically discussed in previous studies.

In the current study, to investigate whether those with clinically node-negative colon cancer found to be pathologically diagnosed with node involvement could be treated with radical surgery alone, only pT1-3N1a colon cancer patients without preoperative identification of lymph node metastases were included into analyses. As this was to our knowledge, the first research to explore this problem, we believed that a large-scale database was the most suitable option as it enabled us to access more data.

According to the patient data included in the study, for those with very low lymphatic tumor burden, the 5-year CSS rates of patients without and with adjuvant chemotherapy were 80.0 and 90.7%, respectively. The results of multivariate Cox regression analyses also showed that the receipt of adjuvant chemotherapy after the radical resection of the primary tumor was independently associated with a 57.3% decreased risk of colon cancer-specific mortality compared with surgery alone. In addition, the 5-year OS rates of patients without adjuvant chemotherapy (52.1%) was lower than patients with adjuvant chemotherapy (82.1%), and the receipt of adjuvant chemotherapy after the radical resection of the primary tumor was also shown to be independently associated with a 64.0% decreased risk of overall mortality compared with surgery alone. All of the above findings demonstrated that adjuvant chemotherapy was significantly associated with improved prognosis and radical surgery alone was not enough in colon cancer with very low lymphatic tumor burden.

In stage III colon cancer, patients were commonly treated with chemotherapy and it was a standard treatment of node-positive disease after the radical resection of the primary tumor, which was reported to result in 8–10% improvement in overall survival (16, 17). However, both patients and medical oncologists should be aware of the toxicity caused by chemotherapy, and it has been reported for example, that patients treated with oxaliplatin might develop late-onset neuropathy with adverse impact on the personal lives and work of patients (18, 19). There is, therefore, a need to stratify stage III colon cancer patients into different prognostic subgroups to generate better therapeutic options for low-risk patients who may not require high dose chemotherapy.

Recently, the IDEA (International Duration Evaluation of Adjuvant therapy) demonstrated that CAPOX (capecitabine and oxaliplatin) treatment for 3 months is as effective as 6 months in ensuring disease-free survival among low-risk (T1-3N1) but not high-risk (T4 or N2) patients. These results have led many medical oncologists to consider 3 months of adjuvant treatment as the new standard of care for low-risk stage III disease, indicating that the standard long-course chemotherapy was not necessary for stage III colon cancer with low lymphatic tumor burden (20, 21).

In 2014, Tashiro et al. (22) examined the efficacy of adjuvant chemotherapy in stage III colon cancer. Although adjuvant therapy raised their chances of survival by three-fold compared with curative surgery alone, this study found that chemotherapy did not affect the 3-year OS and the 3-year RFS of stage IIIA patients. This led them to believe that chemotherapy might be omitted for certain cases of stage IIIA colon cancer with low risk of recurrence, such as node positive T1/T2 patients.

In 2016, Akeel et al. conducted a retrospective analysis with 218 clinically node negative rectal cancer patients undergoing radical surgery using total meso-rectal excision (TME) techniques for rectal cancer with curative intent from 2000 to 2012. These cases were later confirmed to be stage III disease on final pathology from a prospectively maintained database, and they found that TME surgery alone was not sufficient for those patients, which is consistent with our study (23).

In research by Tashiro et al. it was noted that stage IIIA diseases were mixed with non-N1a colon cancer. Moreover, the sample size of stage IIIA colon cancer was too small (22 patients in the adjuvant chemotherapy group and 17 patients in surgery alone group) and the survival differences between the two groups did not reach statistical significance. Therefore, as mentioned above, we strongly believed that adjuvant chemotherapy was significantly associated with improved prognosis, and that radical surgery alone was not enough in colon cancer with very low lymphatic tumor burden.

The main strengths are first, that it was reasonable to ask whether those with clinically node-negative colon cancer found to be pathologically diagnosed with node involvement, could be a favorable subgroup in stage III disease and could they be treated with radical surgery alone to avoid overtreatment, based on previous findings. Second, to the best of our knowledge, this is the first study to examine the necessity of adjuvant chemotherapy in colon cancer patients with a very low lymphatic tumor burden. Third, our study was based on a large population-based using the SEER database, which increased the credibility of the results.

However, the present study also had two weaknesses. First, the chemotherapy regimens and the information of comorbidities were not available in the SEER database. Many previous studies (24–27) have reported that the presence of comorbidities was associated with a decrease in cancer treatment, which would cause a selection bias in the present study. Second, due to its retrospective nature, selection bias may exist in the current study, and results subsequently need to be interpreted cautiously.

## Conclusions

In conclusion, our study retrospectively analyzed the efficacy of adjuvant chemotherapy in patients with clinically node-negative colon cancer found to be pathologically diagnosed with node involvement and demonstrated that adjuvant chemotherapy was significantly associated with improved prognosis and radical surgery alone was not enough in colon cancer with very low lymphatic tumor burden.

## Data Availability Statement

Publicly available datasets were analyzed in this study. This data can be found here: Surveillance, Epidemiology, and End Results (SEER) database (https://seer.cancer.gov/).

## Author Contributions

ZZ and CS participated in the design of the study. GX, YJ, and CF participated in collecting data. GX, YJ, and JY conducted the statistical analysis and wrote the first draft of the manuscript. ZZ and CS reviewed the manuscript. All authors read and approved the final version of the manuscript.

## Conflict of Interest

The authors declare that the research was conducted in the absence of any commercial or financial relationships that could be construed as a potential conflict of interest.
